# Beyond the Parental Generation: The Influence of Grandfathers and Great-grandfathers on Status Attainment

**DOI:** 10.1007/s13524-016-0486-6

**Published:** 2016-07-05

**Authors:** Antonie Knigge

**Affiliations:** Department of Sociology/ICS, Utrecht University, Padualaan 14, 3584 CH Utrecht, The Netherlands

**Keywords:** Social mobility, Status attainment, Multigenerational, Grandfathers, Sibling models

## Abstract

Studies on intergenerational social mobility usually examine the extent to which social positions of one generation determine the social positions of the next. This study investigates whether the persistence of inequality can be expected to stretch over more than two generations. Using a multigenerational version of GENLIAS, a large-scale database containing information from digitized Dutch marriage certificates during 1812–1922, this study describes and explains the influence of grandfathers and great-grandfathers on the occupational status attainment of 119,662 men in the Netherlands during industrialization. Multilevel regression models show that both grandfather’s and great-grandfather’s status influence the status attainment of men, after fathers and uncles are taken into account. Whereas the influence of the father and uncles decreases over time, that of the grandfather and great-grandfather remains stable. The results further suggest that grandfathers influence their grandsons through contact but also without being in contact with them. Although the gain in terms of explained variance from using a multigenerational model is moderate, leaving out the influence of the extended family considerably misrepresents the influence of the family on status attainment.

## Introduction

In a fair and an efficient society, individuals are matched to occupations and their accompanying privileges (such as status and wealth) based arguably on their talent rather than the family into which they were born. Thus, many stratification scholars have studied the extent to which occupational attainment is determined by family background. The vast majority of these studies have looked at how the social position of one generation is influenced by the social position of their parents (Breen and Jonsson 2005; Ganzeboom et al. 1991). However, more recently, some have argued that in order to fully understand the social reproduction of families, it may be important for certain contexts to look beyond parents and to take the extended family into account (Mare 2011).

A growing body of research has examined whether the dominant Markovian parent-offspring approach is adequate, or whether it is necessary to adopt a multigenerational perspective to understand intergenerational social mobility. Nevertheless, the number of studies is limited and restricted mostly to grandfathers (but see Campbell and Lee 2003, 2008, 2011). For occupational social mobility, some studies have found direct net effects of grandparents (Allingham 1967; Beck 1983; Chan and Boliver 2013; Goyder and Curtis 1977; Pohl and Soleilhavoup 1982), but others have reported that grandparents play no part after the role of parents has been accounted for (Erola and Moisio 2006; Warren and Hauser 1997).

Because the results are both limited and mixed, the pervasiveness of the influence of generations more remote than parents is unclear. Partly, this is a descriptive empirical problem: more studies need to be conducted to get a reliable picture. However, this is also an explanatory empirical problem: we need to test the mechanisms thought to underlie multigenerational effects to understand the contexts in which we can expect such effects to be prominent. This article confronts both problems by studying the influence of grandfathers and great-grandfathers on the occupational status attainment of men in the Netherlands in the second half of the nineteenth century and the beginning of the twentieth century.

Two mechanisms have been proposed for the influence of grandparents and great-grandparents (over and above that of parents) on the social positions of their grandchildren and great-grandchildren. One mechanism involves the transfer of resources through socialization, requiring contact between the generations (Bengtson 2001). The other mechanism does not presuppose contact: it involves the transfer of durable resources, which are likely to still exist for subsequent generations to benefit from even if the original holder has passed away (Mare 2011).

However plausible these mechanisms may be, they have hardly been systematically tested (for a notable exception, see Zeng and Xie 2014). Testing the mechanisms is not easy because of several complicating factors. Probably the most important deterrent is that few large-scale data sets cover more than two generations; even fewer also contain detailed information on, for example, contact between grandparents and grandchildren, or the level of durable resources in family lineages. Because the data that I use largely overcome these problems, this study makes substantial headway in testing what I refer to as the “contact mechanism” and the “durable resource mechanism.”

I analyze a large-scale database, GENLIAS, which contains digitized information from Dutch marriage certificates for the period 1812–1922—a period in which just a small percentage of the population did not marry. These marriage records contain information on the occupations of those who married and of their parents. Where possible, the marriage certificates have been linked to the marriage certificates of parents for 5 of 11 provinces. I study only men because the status attainment of women in this time frame was quite different (Bras 2002; Schulz 2013) and deserves a separate study. I also exclude the families-in-law, given that it is unlikely in the context studied that they were willing to invest resources in the groom before marriage (and thus before the measurement of occupational status). Altogether, I am able to apply multilevel sibling models to 43,242 paternal grandfathers, 64,062 of their sons, and 119,662 of their grandsons. For 25,433 men, I can even study the influence of 9,116 great-grandfathers. An advantage of using multilevel models is that they allow studying both conventional measures of family influence (father-son and grandfather-grandson correlations) and what are often regarded as more comprehensive measures of family influence (brother and cousin correlations) (Hällsten 2014; Jencks et al. 1972; Knigge et al. 2014a).

The Netherlands during industrialization forms a fruitful context in which to study multigenerational influence. First, although the Netherlands had its own peculiarities (such as an early developed service sector), it can be considered exemplary for other Western modernizing societies in many respects, including the modernization processes that took place. The present study is the first to provide empirical evidence on whether the conventional two-generation view is adequate to enable an understanding of intergenerational mobility in the context of a modernizing Western society, or whether a multigenerational view seems warranted.

Furthermore, the effects of the two aforementioned mechanisms can be separated to some extent because of the specific characteristics of this period. Durable resources are thought to have been especially relevant for attaining status in the nineteenth century, albeit decreasingly so because of modernization processes. This claim can be tested because for great-grandfathers, contact was virtually impossible given the prevailing life expectancy. Thus, if great-grandfathers had an influence, it must have been through durable resources. The contact mechanism, on the other hand, may have become more important in this period because increasing life expectancy resulted in a longer period of shared lives between grandfathers and grandsons. Although I do not have a direct measure of contact, I can measure the likelihood of contact by looking at whether grandfathers lived near (in time and space) their grandsons.

One final contribution of this study is that it tests to what extent the influence of grandfathers is actually that of uncles. Because this article tests mechanisms for direct effects of grandfathers, teasing out any indirect effects via uncles is important. Some historical studies have suggested that Dutch uncles played a role in the lives of their nephews (Kok and Mandemakers 2010; Kok et al. 2011). On one hand, uncles may have helped their nephews by providing work or resources. On the other hand, the presence of uncles may have meant competitive claims to grandparental resources. By testing whether uncles have an effect, this study will show whether research should start developing and testing theories on the role of uncles (and other extended family members) in the future as well.

## Theoretical Background and Hypotheses

### Influence of Grandfathers and Great-grandfathers on Status Attainment

#### Influence Through Contact

Parents influence the status attainment of their children through the transfer of resources, such as financial, cultural, human, and social capital (Blau and Duncan 1967; Bourdieu and Passeron 1977/1990). Grandparents and great-grandparents can influence the status attainment of their grandchildren/great-grandchildren in the same way by taking over or complementing the parents’ role (Bengtson 2001; Zeng and Xie 2014). For example, grandparents can look after their grandchildren while parents work, or grandparents/great-grandparents can make financial contributions to the cost of educating their grandchildren/great-grandchildren. In the Netherlands in the nineteenth century, it was almost impossible for great-grandfathers to help raise their great-grandsons because the low life expectancy made contact between them unfeasible.

One could argue that grandfathers did not play a central role in the lives of their grandchildren, either. Nuclear families were the standard, with an average household size of approximately 4.8 (Kok and Mandemakers 2010). Most families consisted of a married couple with or without children, and extended-family households were not common.^1^ Moreover, life expectancy was much lower than it is nowadays. Men born in 1820 who reached the age of 30—the age at which they were likely to have their first son (Van Poppel 2012)—were expected to die at age 63.^2^ Thus, many children never knew their grandfathers because grandfathers, on average, would die before or soon after the birth of grandchildren. Because of the limited frequency of extended-family households and the low life expectancy, only about 9 % of children were born into a household with at least one grandparent present; and by age 15, hardly any children lived with their grandparents.

However, it is not unlikely that grandfathers had an impact on their grandsons’ status attainment through direct contact. First, although coresidency was generally not common, most grandparents lived close to their grandchildren (Van Poppel 2012). Furthermore, both life duration and the age at which people had their first child varied greatly: for example, 40 % of men born in 1820 who reached the age of 30 died at the age of 70, and 15 % died at the age of 80 (Van Poppel 2012). Therefore, many grandchildren’s lives did overlap with that of at least one grandparent. Post et al. (1997) estimated that approximately 75 % of children who were aged 0–20 in the period 1850–1900 had at least one grandparent still alive (but fewer than 5 % had all four grandparents still alive). Because of this possibility for contact, I expect to find the following:

- *Hypothesis 1* (H1). Grandfathers’ occupational status positively influenced their grandsons’ occupational status in the Netherlands during modernization.

Transferring resources through contact was difficult if grandparents died soon after their grandchildren were born, and obviously impossible if they died before. Therefore, I expect the opportunities for grandparents to influence their grandchildren through direct contact to be fewer when the overlap of the lives of grandparents and grandchildren is smaller:

- *Hypothesis 2a* (H2a). The positive influence of grandfathers’ occupational status on their grandsons’ occupational status is lower the less that their lives overlap.

If grandfathers live far from their grandsons, it is also more difficult for them to have an influence through direct contact. Geographical distance formed a serious obstacle in the nineteenth century, with the development of mass transportation and mass communication only just starting (Knippenberg and De Pater 2002). This leads to the following hypothesis:

- *Hypothesis 2b* (H2b). The positive influence of grandfathers’ occupational status on their grandsons’ occupational status is lower, the greater the geographical distance between them.

#### Influence Without Contact: Durable Resources

Mare (2011) proposed several arguments regarding how grandparents could influence their grandchildren’s status attainment without being in contact with them. I classify these modes of influence under the heading “durable resource mechanism.” To start, Mare argued that many resources relevant for attaining status are quite durable. Some resources, such as human and cultural capital, which are relatively important for educational attainment, can typically be transferred only as long as the holder is alive. However, other resources, such as financial and physical wealth (e.g., land and property), are much less perishable: that is, such resources may still exist for future generations to benefit from, even if the intermediate generation did not benefit. Such durable resources are expected to have been relatively important in the Netherlands in the nineteenth century because a large share of the population (40.3 % in 1849) was employed in agriculture (Smits et al. 2000), and educational opportunities were still limited (Mandemakers 1996).

Further, Mare (2011) argued that social institutions, too, outlive individuals and may therefore be seen as potential durable resources. Especially at the top and bottom of the hierarchy, social institutions could lead to extreme advantages and disadvantages. As an example of institutionalized advantage, Mare mentioned the university legacy admission systems in the United States, by which grandsons can enter a top university more easily if their grandfather graduated there. This system did not exist in the Netherlands, but the nobility system and the student fraternities (*studenten corpora*) are examples of institutionalized advantage in the Dutch case. Moreover, it is highly possible that informal reputation mechanisms produced similar effects (“I knew your grandfather, he was a great man, and therefore I will help you”). In the absence of diplomas to signal productivity, employers may rely more on the reputation of family lineages. Also, the reputation of successful grandfathers may serve as an inspiration for their grandchildren.

In conclusion, it is highly possible that grandfathers influenced their grandsons through durable resources, providing a second mechanism for H1. Similarly, great-grandfathers can be expected to have had an influence on their great-grandsons through durable resources, but for them, this would have been the only mechanism.

- *Hypothesis 3* (H3). Great-grandfathers’ occupational status positively influenced their great-grandsons’ occupational status attainment in the Netherlands during modernization.

### Changes in the Influence of Grandfathers and Great-grandfathers Over Time

Many scholars have claimed that in Western societies in the past, family background was much more important for attaining status than it is in contemporary societies. The argument is that modernization processes (such as industrialization, educational expansion, and mass communication) rendered ascriptive characteristics (roughly, family background) less decisive and achieved characteristics (roughly, educational attainment) more decisive in the status-attainment process (Blau and Duncan 1967; Kerr et al. 1960; Treiman 1970). On the other hand, status maintenance theory argues that in modernized societies, elites found alternative strategies to transmit status to the next generation: for example, by ensuring that their children received a good education (Bourdieu and Passeron 1977/1990; Collins 1971).

In the Netherlands, the modernization processes discussed by Treiman (1970) occurred in the second half of the nineteenth century. An initial wave of industrialization in the form of mechanization of labor occurred around 1865, and a second, more significant wave occurred in the period 1895–1914 (De Jonge 1968; Van Zanden and Van Riel 2004). Industrialization caused shifts in the proportions of the labor force employed in agriculture, industry, and the service sector. In 1807, 43.1 % of the total labor force was employed in agriculture; 26.2 %, in industry; and 30.8 %, in services. By 1909, these figures were 30.4 %, 34.4 %, and 35.4 %, respectively (Smits et al. 2000).

Knigge et al. (2014b) found, in line with modernization theory but not with status maintenance theory, that the influence of family background on the status attainment of Dutch men declined in the second half of the nineteenth century, and was less where communities were more modernized. If Dutch society did indeed become more open because of modernization, one would expect not only fathers to have had less influence but also grandfathers and great-grandfathers (and uncles) because a change from ascription to achievement meant that the extended family, too, would have been less of a help or a hindrance in attaining status.

**Fig. 1:**
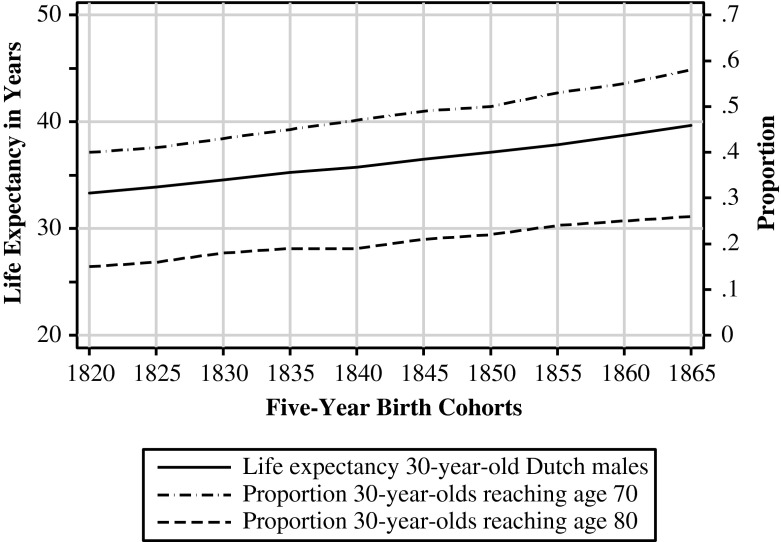
Life expectancy of 30-year-old Dutch males, and the proportion of 30-year-old Dutch males reaching ages 70 and 80 (five-year birth cohorts, 1820–1865). *Source:* Generation life tables (*generatie-sterftetafels*) from Statistics Netherlands (CBS)

However, we must take into account another development before formulating hypotheses. Evidence suggests that the lives of grandfathers and grandsons overlapped more over time. Figure 1 shows that life expectancy at age 30 rose steadily, from 33 years for men born in 1820 to 37 years for men born in 1850.^3^ Also, the percentage of 30-year-old men living at least another 40 years increased in the same period, from 40 % to 50 %. Although this evidence is far from conclusive, it suggests that the opportunities grandfathers had to influence their grandsons through contact increased over time, which would have counteracted the trend resulting from the lessened importance of (durable) family resources attributable to modernization. Because there is no convincing argument regarding which of the opposing developments had the most impact on the influence of the grandfather, it seems appropriate to expect no change in grandfather influence over time. Because great-grandfathers were unable to influence through contact but only through durable resources, the influence of great-grandfathers is expected to have declined over time.

- *Hypothesis 4* (H4). The positive influence of grandfathers’ occupational status on their grandsons’ occupational status remained stable during modernization in the Netherlands.
- *Hypothesis 5* (H5). The positive influence of great-grandfathers’ occupational status on their grandsons’ occupational status declined during modernization in the Netherlands.

## Method

### Data

I use the data base GENLIAS (version 2007_03), which contains digitized information from Dutch marriage certificates for the period 1812–1922. A marriage certificate typically states date and place of marriage; names, place of birth, age, and occupation of the bridegroom and bride; and names and occupations of the couple’s parents. For the provinces Groningen, Overijssel, Gelderland, Limburg, and Zeeland, the marriage certificates have been linked to the marriage certificates of the parents. A computer algorithm matched the first and last names of the parents as stated on both certificates. To avoid incorrect links, the computer algorithm used additional information, such as the age of the bride and groom, to ensure plausibility in terms of chronology (for more details, see Oosten 2008).

From this database, I created a three- and four-generation version by matching the entries in which an individual is a groom in one and the father of the groom in another. Further filtering as well as deleting cases with missing data (see the following section) yield 43,242 grandfathers married between 1812 and 1881 on whose married sons and grandsons I have data. Put otherwise, I can identify the father, paternal grandfather, uncles (father’s married brothers), brothers, and cousins of 119,662 men married between 1854 and 1922. For 25,443 grooms, I can perform analyses that include 9,116 great-grandfathers.

#### Selections and Missing Data

As discussed in the Introduction, I study neither women nor the families-in-law. Also, I include only men marrying for the first time because I want to ensure that each person appears in the database only once and because family influence might work differently when an individual marries for the second time. This results in a database of 952,587 grooms married between 1812 and 1922. The marriage certificates of 526,119 of these grooms could be linked to the marriage certificate of the father. In turn, in 248,777 of these cases, the marriage certificate of the father could be linked to that of his father (the grandfather). In 67,964 of these cases, we also know the great-grandfather.

**Fig. 2:**
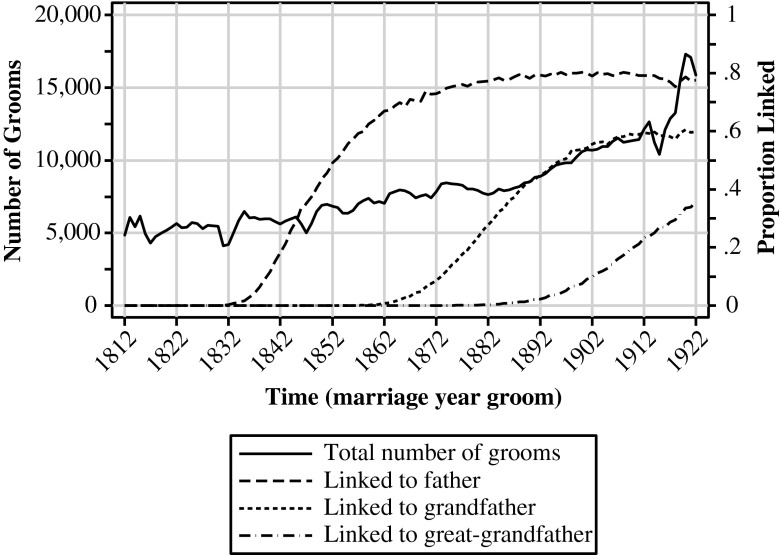
Number of grooms and proportion linked per year

A significant proportion of the marriage certificates cannot be linked for several reasons. First, the fathers of grooms who married shortly after 1812 will certainly have married before 1812 and will not be part of the database. None of the grooms who married before 1831 can be linked to their father. The same issue occurs in linking fathers’ certificates to grandfathers’ certificates, and linking grandfathers to great-grandfathers. The earliest date for which I can link a groom (via the father) to his grandfather is when the groom married in 1854; and the earliest date for which I can link a groom to his great-grandfather is when the groom married in 1871. Figure 2 exhibits the number of grooms that married in each year, as well as the proportion of these grooms that could be linked to their father, grandfather, and great-grandfather, respectively. The proportion linked to their grandfather remains less than .01 until 1862 but then increases to become .60 in 1922 (the average for the period 1854 to 1922 is .38). The proportion linked to their great-grandfather remains less than .01 until 1887 and reaches .36 in 1922 (the average for the period 1871 to 1922 is .13). Keep in mind when interpreting the results that the proportion of successful links is thus limited, especially in the first few years after 1854 and 1871.

Second, individuals could be linked only within and between 5 of 11 provinces, so grooms could not be linked where the father or grandfather/great-grandfather had married outside these five provinces. However, I do not expect the proportion that could not be linked for this reason to be very large, as I explain in the next section.

Third, variation in the spelling of names may result in failure to establish a link. The computer algorithm was designed to allow for minor variations in the spelling of names. However, a conservative approach was taken in this respect to minimize the number of incorrect links at the expense of not maximizing the number of total links. Finally, nonlinkage may result from errors in digitizing the certificates.

Cases cannot be analyzed when the certificates lack occupational data sufficient to assign a status score to grooms (1.44 % of the cases in the three-generation data set; 1.14 % of the cases in the four-generation data set), to fathers (19.4 %; 15.9 %), grandfathers (23.6 %; 21.3 %), uncles (28.2 %; 30.2 %), and great-grandfathers (N.A.; 25.6 %). Listwise deletion of these cases (51.9 %; 62.6 %) results in the 119,662 and 25,443 grooms mentioned earlier.

#### Reflection on Possible Selection Bias in the Data

These data provide a rare opportunity to study multigenerational processes over an extensive period while covering a broad geographical area. Nevertheless, like most historical data, they have certain drawbacks. An obvious limitation of using marriage certificates is the exclusion of people who never married. This exclusion is less problematic than might be expected because marriage was common in the Netherlands in the nineteenth and early twentieth centuries: approximately 87 % of all men born in 1800 and 91 % of all men born in 1900 married at some point (Ekamper et al. 2003). Furthermore, Engelen and Kok (2003) did not find many significant differences (in terms of family background, religion, region, and birth cohort) in the likelihood of men born between 1890 and 1909 remaining unmarried. Schulz (2013) found no significant difference in status between married and unmarried Dutch men during the period that she studied (1865 to 1930).

Because records were linked within and between 5 of 11 provinces, I lose grooms if they, their fathers, or paternal grandfathers migrated from the region; and I lose family members if grooms, their fathers, or paternal grandfathers migrated to the region. Migrants are not a random selection given that they tend to have a higher status, but I do not believe this will influence the results substantially, for two reasons. First, the number of people I miss because of migration is not very large. Census data show that in 1849, just 8 % of people lived in a province other than the one in which they were born; the corresponding figures for 1899 and 1930 were 13 % and 15 %, respectively (Knippenberg and De Pater 2002). Furthermore, the data set does include those who migrated between the five provinces in the data, or who moved away after marrying. Second, Knigge et al. (2014b) performed several checks on the same data and showed that the effect of family influence on status attainment changes little when including less or more migrants.

Finally, marriage certificates frequently lack information on the father’s occupation. Linking the data alleviates this problem because the marriage certificates of siblings can be used as sources of information on the father’s occupation (for fathers, the problem is reduced from 32.7 % to 19.4 % of cases). Still, because the problem affects grandfathers, great-grandfathers, and uncles as well, the combined number of missing cases is considerable. If a father’s occupation is missing on his child’s marriage certificate, the most likely reason is that the father was deceased; other explanations include migration or unemployment. Fortunately, in line with other studies (Maas et al. 2011; Zijdeman 2010), I find little difference between those with and those without information on the father’s occupation. For example, occupational status differs by less than 1 point on an 88-point scale (47.28 and 48.25, respectively), and the status correlation between brothers is also rather similar (0.51 and 0.54, respectively). Moreover, the father-son correlation is not substantially different for those with and those without information on the grandfather’s occupation (0.52 and 0.54, respectively).

### Measures

#### Dependent and Independent Variables

**Fig. 3:**
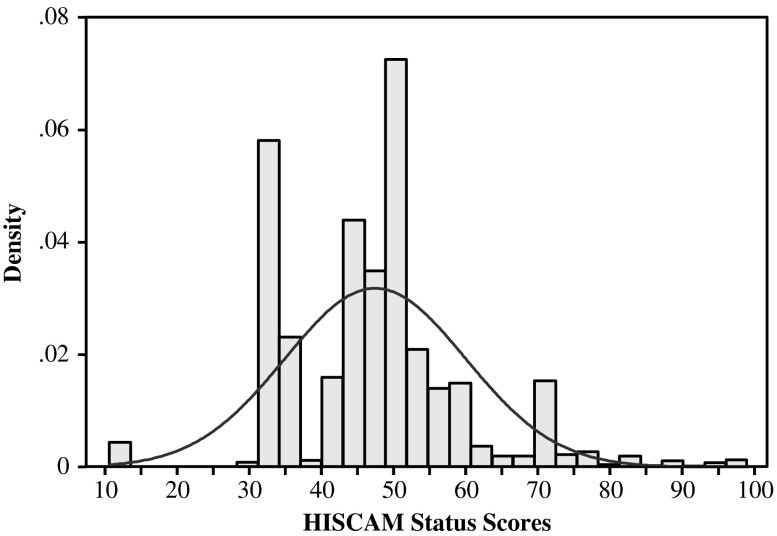
Histogram of the occupational status of Dutch men married between 1854 and 1922

Occupations have been coded using the Historical International Standard Classification of Occupations (Van Leeuwen et al. 2002), which is the historical equivalent of the International Labour Organization’s International Standard Classification of Occupations (ISCO68). These occupational codes were subsequently mapped onto the HISCAM status scale (Lambert et al. 2013), which uses the same technique as the contemporary CAMSIS status scales (Stewart et al. 1980). In theory, the HISCAM scale runs from 1 to 99; in practice, however, it runs from 10.6 (servant) to 99 (judge, for example). The *occupational status of the youngest generation*—the dependent variable—is based on the occupations stated on the marriage certificate. Table 1 provides descriptive information on all variables (separately for the three- and four-generation data sets). The histogram in Fig. 3 gives more detail on the distribution of grooms’ occupational status, showing that it approximates the normal distribution but with a few spikes for frequent occupations, such as worker (32.5) and farmer (50.7).

**Table 1:** Descriptive information variables

	Analyses Without Great-grandfathers				Analyses With Great-grandfathers			
	Mean	SD	Min.	Max.	Mean	SD	Min.	Max.
Variables								
Occupational status son	47.16	12.26	10.60	99.00	47.64	12.51	10.60	99.00
Occupational status father	46.70	9.89	10.60	99.00	46.90	10.29	10.60	99.00
Occupational status grandfather	45.19	9.11	10.60	99.00	44.90	9.06	10.60	99.00
Occupational status great-grandfather					44.56	8.69	10.60	98.40
Average occupational status uncles	46.81	9.67	10.60	99.00	47.04	10.06	10.60	99.00
Time	5.00	1.31	0.00	6.80	4.19	0.77	0.00	5.10
Temporal distance	66.84	9.50	38.00	125.00	62.19	7.64	38.00	95.00
Geographical distance (ln)	1.55	1.51	0.00	6.48	1.50	1.45	0.00	5.74
Control Variables								
Age at marriage	26.48	4.72	16.00	69.00	25.19	3.79	16.00	55.00
Birth order	2.58	1.67	1.00	14.00	2.41	1.59	1.00	13.00
Sibship size	4.19	2.09	1.00	14.00	3.87	2.05	1.00	13.00
Father farmer	0.30		0.00	1.00	0.26		0.00	1.00
Number of uncles and aunts	3.33	2.17	0.00	14.00	3.33	2.20	0.00	12.00
Grandfather farmer	0.31		0.00	1.00	0.29		0.00	1.00
Having no uncles	0.30		0.00	1.00	0.30		0.00	1.00
Great-grandfather farmer					0.31		0.00	1.00
Number of Individuals			119,662				25,443	
Number of Fathers			64,062				14,547	
Number of Grandfathers			43,242				10,142	
Number of Great-grandfathers							9,116	
Number of Communities			16,142				5,343

*Father’s occupational status* is the average status of the occupations that he reported on his children’s marriage certificates. The reliability of this group-averaged score can be calculated using the Spearman-Brown prediction formula (Winer et al. 1991: appendix E) and is estimated by Stata’s *loneway* command to be .875 for the average-sized family. Thus, the intergenerational correlations will be slightly underestimated. The *occupational status ofgreat-grandfathers, grandfathers, and uncles* are similarly derived from their children’s marriage certificates. Because a groom may have more than one uncle, I take the mean of all married uncles. Moreover, to prevent losing cases, I substitute the father’s occupational status for those who do not have an uncle (and adjust for this in the analyses; see the Control Variables subsection).

*Time* is operationalized as the marriage year of the grandson/great-grandson. I rescale by subtracting the first year (1854 for analyses without the great-grandfather; 1871 for analyses with the great-grandfather) and then dividing by 10.

To approximate whether a grandfather influenced a grandson directly through contact, I use two indicators for the likelihood that they were in contact. *Temporal distance* is given by the age difference between grandfather and grandson. I assume that the smaller the age difference, the greater the chance that grandfather and grandson had overlapping lives. *Geographical distance* is given by the distance in kilometers between the grandfather’s place of marriage and the grandson’s place of marriage. Because this measure would be right-tailed, I take the natural log (after adding 1). I assume that the smaller the geographical distance between grandfather and grandson, the greater the chance that they were in contact.

#### Control Variables

I include several control variables that might be confounding factors (e.g., Bras et al. 2010). At the individual level, these are the *age at marriage* of the groom as found on his marriage certificate, and *birth order*, the birth rank of a groom among his married siblings. At the family level, this is *sibship size*, which is approximated by the number of married full brothers and sisters; and a dummy variable representing whether the *father was a farmer* (1) or not (0) (cf. Erikson and Goldthorpe 1992): a father is labeled a farmer if more than one-half of his children providing information about their father’s occupation state that he is a farmer (HISCO codes 61110 to 61290). At the extended-family level, this is the *number of married uncles and aunts* (as they and their children may be competitors for grandparental resources) and whether the *grandfather/great-grandfather was a farmer* (constructed in the same way as for the father).^4^ Finally, to correct for substituting uncles’ status with father’s status for *grooms without any uncles*, I include a dummy variable representing whether a groom has at least one uncle (0) or no uncles (1). More importantly, I include an interaction of this dummy variable with the variable status of uncles to ensure that the coefficient of status of uncles reflects only the effect for those who have an uncle (one would expect the effect for those without uncles to be insignificant). For the same reason, a three-way interaction with the dummy variable is included if the status of uncles is interacted with time in the analysis.^5^

### Analytical Strategy

I perform multilevel linear regression with four hierarchical levels (individuals, fathers, grandfathers, and communities)^6^ using the package that runs MLwiN from within Stata (Leckie and Charlton 2013; Rasbash et al. 2013). I include communities as a fourth level because individuals growing up in the same time period and the same geographical area tend to resemble one another. In reality, brothers and especially cousins might grow up in different communities, but the cross-classified models that would do justice to this structure are too complex to estimate. Therefore, I simplify and keep the multilevel structure hierarchical by defining the community as the marriage year and marriage place of the grandfather.

To describe how large the influence of the family and extended family is on occupational status attainment, I start by estimating the intercept-only model:

M1 $$ {Y}_{ijkl}={\upbeta}_{0000}+{c}_{0l}+{g}_{0 kl}+{f}_{0jkl}+{s}_{0 ijkl}, $$

where *Y*_*ijkl*_ is the occupational status of individual *i* with father *j* and grandfather *k* from community *l*, β_0000_ is the population mean status, ${c}_{0l}\sim \left(0,{\upsigma}_{c_{0l}}^2\right)$ is the error term at the community level, ${g}_{0 kl}\sim \left(0,{\upsigma}_{g_{0 kl}}^2\right)$ is the error term at the grandfather level, ${f}_{0jkl}\sim \left(0,{\upsigma}_{f_{0jkl}}^2\right)$ is the error term at the father level, and ${s}_{0 ijkl}\sim \left(0,{\upsigma}_{s_{0 ijkl}}^2\right)$ is the error term at the individual level (Snijders and Bosker 1999).^7^ The proportion of variance at the father, grandfather, and community levels is given by

1 $$ {\uprho}_{c+g+f}=\frac{\upsigma_{c_{0l}}^2+{\upsigma}_{g_{0 kl}}^2+{\upsigma}_{f_{0jkl}}^2}{\upsigma_{c_{0l}}^2+{\upsigma}_{g_{0 kl}}^2+{\upsigma}_{f_{0jkl}}^2+{\upsigma}_{s_{0 ijkl}}^2}, $$

which is the expected correlation between two randomly selected brothers. This brother correlation is often considered a comprehensive measure of family impact because it captures all the aspects of family background that siblings share (Björklund et al. 2009), including not only all (measurable and nonmeasurable) shared family resources but also, for example, shared neighborhood characteristics and brothers’ influence on one another (Jencks et al. 1972). Given that cousins have the same grandfather (and the same community because of the mentioned modeling simplification) but not the same father, the expected correlation between two randomly selected cousins is given by

2 $$ {\uprho}_{c+g}=\frac{\upsigma_{c_{0l}}^2+{\upsigma}_{g_{0 kl}}^2}{\upsigma_{c_{0l}}^2+{\upsigma}_{g_{0 kl}}^2+{\upsigma}_{f_{0jkl}}^2+{\upsigma}_{s_{0 ijkl}}^2}. $$

The observed values for these measures can be compared with what would be expected if intergenerational status transmission followed a Markovian pattern (i.e., one generation was directly influenced only by the previous generation and not by more remote generations).

Another way to assess whether a two-generation model adequately represents family influence is to add status measures of the (extended) family. In Model 2,

M2 $$ {Y}_{ijkl}={\upbeta}_{0000}+{\upbeta}_{0100} FSTA{T}_j+{c}_{0l}+{g}_{0 kl}+{f}_{0jkl}+{s}_{0 ijkl}, $$

the regression coefficient β_0100_ shows the extent to which the father’s occupational status contributes to attaining status. I subsequently add the status of the grandfather (+β_0010_*GSTAT*_*k*_) in Model 3 and the average status of uncles (+β_0200_*USTAT*_*j*_) in Model 4 to see whether they have an effect over and above that of the father (H1).^8^ I add controls in Model 5. Model 6 shows how the three family effects change over time (H4) by including the following interactions with time:

$$ \left(+{\upbeta}_{1100} FSTA{T}_{jkl}TIM{E}_{ijkl}+{\upbeta}_{1010} GSTA{T}_{kl}TIM{E}_{ijkl}+{\upbeta}_{1200} USTA{T}_{jkl}TIM{E}_{ijkl}\right). $$

To test the contact mechanism, I add the following interactions of temporal distance (H2a) and geographical distance (H2b) with the grandfather’s status in Model 7:

$$ \left(+{\upbeta}_{2010} GSTA{T}_{kl}TDI{S}_{ijkl}+{\upbeta}_{3010} GSTA{T}_{kl}GDI{S}_{ijkl}\right). $$

Finally, to test the durable resource mechanism, I analyze the subset of cases that can be linked to their great-grandfather. I start in Model 8 by estimating an intercept-only model similar to Model 1, except that there is now an additional great-grandfather level, ${h}_{0 lm}\sim \left(0,{\upsigma}_{h_{0 lm}}^2\right)$, and—to keep a hierarchical structure—the community level is defined as the marriage year and the place of the great-grandfather instead of the grandfather. Model 9 includes controls and the status measures of father, grandfather, and uncles. Model 10 includes the status of the great-grandfather to see whether he has an additional influence (H3), and Model 11 tests whether this influence declines over time, as expected (H5).

## Results

### Influence of Father, Grandfather, and Uncles on Occupational Status Attainment

#### Family Influence: Status Resemblance of Brothers and Cousins

Model 1 in Table 2 shows that for the Netherlands, in the second half of the nineteenth and the early twentieth century, the status resemblance of brothers—the comprehensive measure for family impact—is ρ_*c* + *g* + *f*_  = .502 (Eq. (1)).^9^ Also, male cousins are rather similar in status (ρ_*c* +*g*_ = .321; Eq. (2)), even though they are much more “remote” family than brothers. These results are not congruent with the Markovian model, in which individuals are influenced only by their parents. A correlation between the status of father and son of .7 would produce the observed fraternal resemblance of about .49 (.7 × .7). In a Markovian world, the expected correlation between the statuses of grandfather and grandson would then also be .49, and that of cousins would be .24 (.49 × .49). The latter is much lower than the observed correlation between cousins (.321), perhaps because the process of status attainment is influenced not only by the parents but also by the grandparents.^10^

**Table 2:** Influence of occupational status of fathers, grandfathers, and uncles on occupational status of men married in the Netherlands between 1854 and 1922

	Model 1	Model 2	Model 3	Model 4
Fixed Part^a^				
Intercept	47.300***	47.178***	47.188***	47.191***
	(0.058)	(0.042)	(0.041)	(0.041)
Status father^b^		0.640***	0.564***	0.520***
		(0.004)	(0.004)	(0.004)
Status grandfather^b^			0.177***	0.143***
			(0.005)	(0.005)
Status uncles (average)^b^				0.105***
				(0.005)
Random Intercepts				
${\upsigma}_{c_{0l}}^2$ (community level)	12.796***	4.530***	4.208***	3.936***
	(0.570)	(0.282)	(0.270)	(0.265)
${\upsigma}_{g_{0 kl}}^2$ (grandfather level)	36.268***	8.423***	8.202***	8.475***
	(0.851)	(0.528)	(0.514)	(0.511)
${\upsigma}_{f_{0jkl}}^2$ (father level)	27.534***	19.539***	18.228***	17.646***
	(0.716)	(0.594)	(0.580)	(0.575)
${\upsigma}_{s_{0 ijkl}}^2$ (individual level)	76.119***	75.678***	75.662***	75.631***
	(0.445)	(0.433)	(0.432)	(0.432)

^a^ Standard errors are shown in parentheses.

^b^ Centered on the mean.

***p < .001

#### Family Influence: Status Measures of the (Extended) Family

Model 2 shows that men profit greatly from having a father with a high status: if father A has 10 status points more than father B, the son of father A is expected to have about 6.4 status points more than the son of father B (*b*_0100_ = 0.640; *p* < .001). By including the father’s occupational status, we can understand much of the impact that the family has. The variance that brothers share $\left({\upsigma}_{c_{0l}}^2+{\upsigma}_{g_{0 kl}}^2+{\upsigma}_{f_{0jkl}}^2\right)$ is reduced from 76.6 in Model 1 to 32.5 in Model 2—a reduction of 57.6 %. The largest proportions of explained variance are at the grandfather (76.8 %) and community levels (64.6 %), indicating compositional effects: communities and grandfathers tend to produce fathers with similar status.

Based on Model 3, I conclude that grandfathers have an influence on the status attainment of men over and above that of fathers (*b*_0010_ = 0.177; *p* < .001). By including the grandfather’s occupational status, the effect of the father is reduced from 0.640 in Model 2 to 0.564 in Model 3. In other words, part of the effect attributed to the father is actually an effect of the grandfather. The net benefits of having a grandfather with a high status are about one-third of the benefits of having a father with a high status. Although the effect of the grandfather is substantial, it does not help explain much better the variation in status attainment: in Model 3, 60 % of the variance shared by brothers is explained, only 2.4 % more than in Model 2. One reason, as shown earlier, is that if the occupational status of the grandfather is omitted, the father assumes part of the effect of the grandfather.

In the next step, I include the average status of the father’s brothers to examine whether grandfathers still have a net effect after the inclusion of uncles. Model 4 shows that the effect of the grandfather’s occupational status declines from 0.177 to 0.143 but remains significant (*p* < .001). This finding means that (1) grandfathers have a direct influence on the status attainment of their grandsons, in line with H1; and (2) 19.2 % of the grandfather effect found in Model 3 is an indirect effect: grandfathers influence their own sons (i.e., sons other than the father), who in turn influence their nephews. The average status of the uncles has a significant positive effect (*b*_0200_ = 0.105, *p* < .001) that is about one-fifth of the father’s effect. Again, the increase in explained shared variance is slight, at only 0.8 %. The effects of the extended family remain after adding controls in Model 5 (see Table 3: if anything, the effects increase).

**Table 3:** Influence of occupational status of extended family on occupational status of men married in the Netherlands between 1854 and 1922, further specified

	Model 5		Model 6		Model 7	
Fixed Part^a^						
Intercept	43.619***	(0.152)	43.664***	(0.152)	42.884***	(0.160)
Status father	0.529***	(0.005)	0.602***	(0.018)	0.597***	(0.018)
× Time			–0.014***	(0.003)	–0.015***	(0.003)
Status grandfather^b^	0.162***	(0.005)	0.160***	(0.017)	0.165***	(0.018)
× Time			–0.000	(0.003)	0.002	(0.003)
× Temporal distance					–0.001*	(0.000)
× Geographical distance					–0.011***	(0.002)
Status uncles (average)^b^	0.111***	(0.005)	0.172***	(0.019)	0.170***	(0.019)
× Time			–0.012**	(0.004)	–0.012***	(0.004)
× No uncles	–0.055***	(0.008)	–0.130***	(0.029)	–0.126***	(0.028)
× Time × No uncles			0.015**	(0.005)	0.014*	(0.005)
Time	0.852***	(0.028)	0.846***	(0.028)	0.800***	(0.029)
Temporal distance^b^					0.028***	(0.004)
Geographical distance (ln)					0.611***	(0.021)
No uncles	0.284**	(0.092)	0.272**	(0.092)	0.287**	(0.092)
Number of uncles and aunts^b^	–0.046*	(0.020)	–0.048*	(0.020)	–0.064**	(0.020)
Grandfather farmer	–0.737***	(0.096)	–0.718***	(0.096)	–0.736***	(0.096)
Sibship size^b^	–0.476***	(0.020)	–0.477***	(0.020)	–0.439***	(0.020)
Father farmer	–2.174***	(0.091)	–2.196***	(0.091)	–2.027***	(0.091)
Age at marriage^b^	0.186***	(0.007)	0.187***	(0.007)	0.179***	(0.007)
Birth order^b^	0.311***	(0.022)	0.308***	(0.022)	0.214***	(0.024)
Random Part						
${\upsigma}_{c_{0l}}^2$ (community level)	2.478***	(0.227)	2.458***	(0.227)	2.813***	(0.232)
${\upsigma}_{g_{0 kl}}^2$ (grandfather level)	7.708***	(0.486)	7.710***	(0.486)	7.523***	(0.481)
${\upsigma}_{f_{0jkl}}^2$ (father level)	16.899***	(0.555)	16.882***	(0.555)	16.559***	(0.550)
${\upsigma}_{s_{0 ijkl}}^2$ (individual level)	74.119***	(0.422)	74.093***	(0.422)	73.557***	(0.419)

^a^ Standard errors are shown in parentheses.

^b^ Centered on the mean.

*p < .05; **p < .01; ***p < .001

In conclusion, leaving out the grandfather’s and uncles’ occupational status would overestimate the effect of the father by 23.1 % (0.640 instead of 0.520), and if I were to base statements about the influence of the family solely on father’s occupational status, as is often done, I would substantially underestimate the family influence compared with statements based also on the occupational status of the extended family. One additional status point for everybody in the extended family would have a combined effect of (0.520 + 0.143 + 0.105) = 0.768, which is 20 % higher than the family effect in the parent-offspring model (0.640). Thus, although men benefit most from having a father with a high status, the status of their grandfather and uncles substantially helps (or hinders) their own social position, too.

#### Influence of the (Extended) Family Over Time

**Fig. 4:**
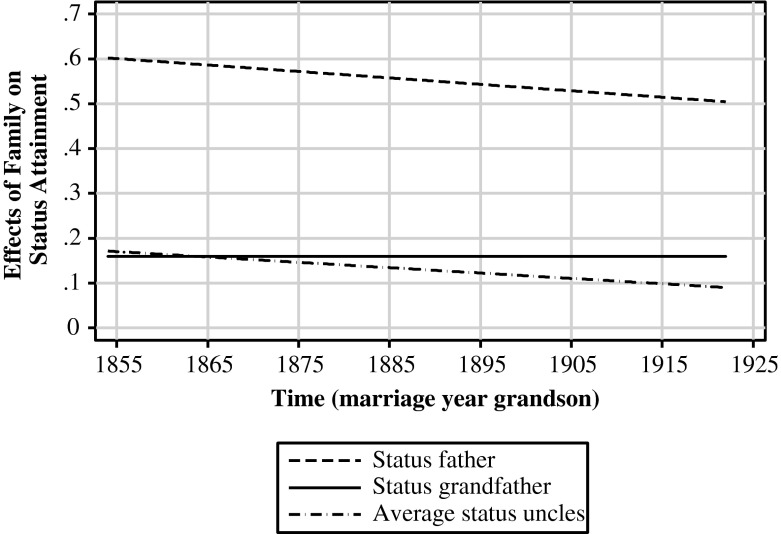
Influence of status of fathers, grandfathers, and uncles over time

In line with the modernization thesis and previous findings, the effect of the father decreased during the nineteenth and early twentieth centuries (*b*_1100_ = –0.014 per 10 years; *p* < .001; see Model 6 in Table 3). A new finding, again consistent with modernization theory, is that the effect of uncles, too, decreased during modernization (*b*_1200_ = –0.012 per 10 years; *p* < .01). I expected that the expanding role of grandfathers in the lives of their grandsons compensated for the effect of modernization (see H4). Indeed, the effect of the grandfather did not decrease but remained constant (*b*_1010_ = –0.000, n.s.). Figure 4 graphs the changes in the (extended) family effects. The influence of the father’s occupational status is approximately 0.6 for men who married in 1854 and approximately 0.5 for men who married in 1922, which shows a decrease of 16.7 % in 67 years. The influence of the uncles reduced by almost one-half (0.09) of what it was (0.17). When the effects of the father, grandfather, and uncles were summed, the family influence decreased 18.3 %, from 0.93 in 1854 to 0.76 in 1922.^11^

### Multigenerational Influence Through Direct Contact

**Fig. 5:**
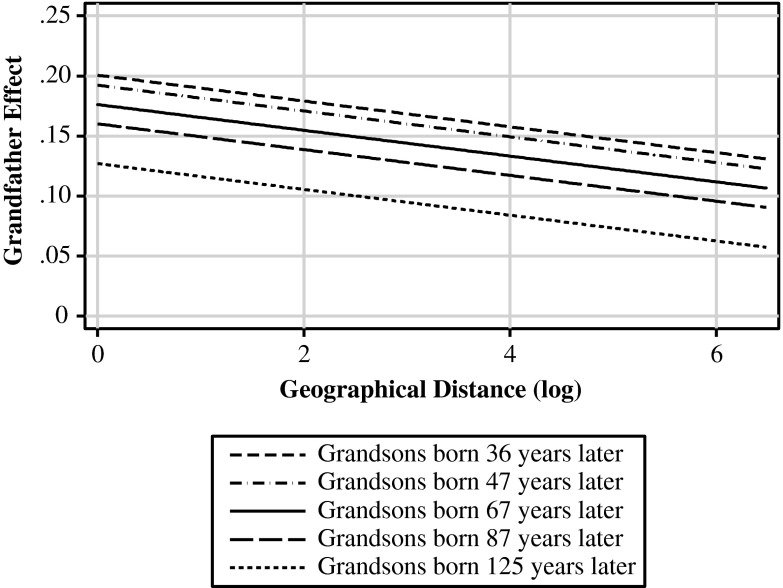
Influence of status of grandfathers by temporal and geographical distance

How did grandfathers influence the status attainment of their grandsons? The most obvious mechanism is through direct contact, by which resources can be passed on directly. I predicted that if this mechanism was at work, the grandfather effect would decline with the lower likelihood of direct contact between the grandfather and his grandson(s), that is, when the temporal (H2a) and geographical distance (H2b) between them increased. Model 7 supports both these predictions: the grandfather effect becomes smaller with increasing temporal distance (*b*_2010_ = –0.001; *p* < .05) and geographical distance (*b*_3010_ = –0.011; *p* < .001). Figure 5 plots the grandfather effect against geographical distance (for those married in 1904, the mean marriage year) for five values of temporal distance: (1) the minimum value (grandson born 36 years after his grandfather), (2) two standard deviations below average (born about 47 years later), (3) average (67 years), (4) two standard deviations above average (87 years), and (5) the maximum value (125 years). The graph shows that if the temporal distance increases, the predicted grandfather effect starts to move toward 0 but never reaches 0. With respect to geographical distance, the graph shows that the grandfather effect is about 0.04 (26.7 %) higher for those grandfathers and grandsons who married in the same municipality (value 0 in the graph) than for those who married about 50 km apart (approximately value 4 in the graph; 95 % of the cases married within 50 km of each other). Taken together, the grandfather effect is predicted to be 0.20 for those most likely to be in contact (temporal distance = 36 years; geographical distance = 0 km) and approximately 0.06 for those for whom it was practically impossible to be in contact (temporal distance = 125; geographical distance = *e*^6^ ≈ 400 km). This large difference is evidence that in the nineteenth century, Dutch grandfathers influenced their grandsons’ status attainment through direct contact. That the effect never becomes 0 may indicate that grandfathers can also have an influence without necessarily being in direct contact with their grandsons.

### Multigenerational Influence Without Contact: The Influence of Great-grandfathers

To further examine the idea that one generation can influence another without direct contact, I test for a subset of the data whether great-grandfathers have an influence (given that it was more or less impossible for them to have been in contact with their great-grandchildren). Because those who can be linked to their great-grandfather may form a special selection, I first check whether results for the subset differ in any way from the results presented for all cases. Model 8 in Table 4 shows that the brother correlation is virtually the same (ρ_*c* + *h* + *g* + *f*_  = .503) as that found in Model 1 (ρ_*c* + *g* + *f*_  = .502). Also, the (extended) family effects are of the same order (Model 9 versus Model 5), lending confidence that the results presented next are not biased by the selection of those who could be linked to great-grandfathers.

**Table 4:** Influence of occupational status of great-grandfather on occupational status of men married in the Netherlands between 1871 and 1922

	Model 8		Model 9		Model 10		Model 11	
Fixed Part^a^								
Intercept	48.087***	(0.119)	44.282***	(0.472)	44.387***	(0.471)	44.378***	(0.472)
Status father^b^			0.511***	(0.010)	0.506***	(0.010)	0.506***	(0.010)
Status grandfather^b^			0.192***	(0.010)	0.158***	(0.011)	0.158***	(0.011)
Status uncles (average)^b^			0.096***	(0.010)	0.089***	(0.010)	0.089***	(0.010)
× No uncles			–0.052**	(0.017)	–0.051**	(0.016)	–0.051**	(0.016)
Status great-grandfather^b^					0.092***	(0.011)	0.128**	(0.044)
× Time							–0.008	(0.010)
Time			0.997***	(0.108)	1.013***	(0.108)	1.015***	(0.108)
Great-grandfather farmer					–0.742***	(0.217)	–0.740***	(0.217)
No uncles			0.348	(0.203)	0.324	(0.203)	0.325	(0.203)
Number of uncles and aunts^b^			–0.105*	(0.044)	–0.101*	(0.044)	–0.101*	(0.044)
Grandfather farmer			–1.012***	(0.212)	–0.797***	(0.233)	–0.795***	(0.233)
Sibship size^b^			–0.538***	(0.050)	–0.529***	(0.050)	–0.528***	(0.050)
Father farmer			–2.718***	(0.205)	–2.681***	(0.206)	–2.685***	(0.206)
Age at marriage^b^			0.317***	(0.019)	0.313***	(0.019)	0.313***	(0.019)
Birth order^b^			0.268***	(0.055)	0.263***	(0.055)	0.261***	(0.055)
Random Part								
${\upsigma}_{c_{0 lm}}^2$ (community level)	14.563***	(1.573)	3.578***	(0.709)	3.267***	(0.658)	3.257***	(0.658)
${\upsigma}_{h_{0 lm}}^2$ (great-grandfather level)	16.762***	(3.173)	0.104	(1.744)	0.000	(0.000)	0.000	(0.000)
${\upsigma}_{g_{0klm}}^2$ (grandfather level)	19.830***	(3.156)	8.349***	(1.931)	8.262***	(1.192)	8.258***	(1.192)
${\upsigma}_{f_{0 jklm}}^2$ (father level)	29.010***	(1.687)	16.502***	(1.293)	16.552***	(1.292)	16.572***	(1.292)
${\upsigma}_{s_{0 ijklm}}^2$ (individual level)	79.322***	(1.044)	77.029***	(0.982)	77.031***	(0.982)	77.022***	(0.982)

^a^ Standard errors are shown in parentheses.

^b^ Centered on the mean.

*p < .05; **p < .01; ***p < .001

Model 10 shows that in line with H3, great-grandfathers have a significant positive effect (*b* = 0.092; *p* < .001) on status attainment, independent of fathers, grandfathers, and uncles. This finding supports the idea that a certain generation may influence subsequent generations “well beyond the grave” because durable resources and certain institutions do not cease to exist after a generation passes away. If great-grandfathers are able to influence their great-grandchildren without being in contact, grandfathers must also be able to influence their grandsons without contact. Furthermore, Model 8 shows that the status resemblance of second cousins (i.e, those sharing the same great-grandfather but a different grandfather) is ρ_*c* + *h*_ = .196. This result is 66.1 % higher than the expected correlation between second cousins if status transmission were to follow a two-generation Markovian process: .7^3^ × .7^3^ = .118 (.7^3^ is the expected correlation between great-grandson and great-grandfather given a father-son correlation of .7, which is deduced from the observed correlation between brothers: .7 × .7 ≈ .5).

I expected that the importance of durable resources and institutions that promote multigenerational influences would have declined with modernization. Therefore, I predicted that the possibility for great-grandfathers to influence their great-grandchildren also decreased as modernization proceeded (H5). Although I find that the great-grandfather effect diminished, this change is not significant (*b* = –0.008, n.s.; see Model 11). This finding could mean that influence without contact did not lose importance in the period studied, but alternatively that the period of observation is too short (see Fig. 2). Indeed, the literature suggests that fairly long periods are necessary in order to detect trends in social mobility (Breen and Luijkx 2004; Ganzeboom et al. 1989).

## Conclusion and Discussion

Studies in the field of intergenerational social mobility usually take a two-generation approach: the influence of the family on status attainment is equated with the influence of the parents. The first aim of this article was to study whether this assumption is justified in the context of a modernizing Western society. Specifically, I studied whether taking a multigenerational perspective by including grandfathers and great-grandfathers leads to a more accurate understanding of the occupational status attainment process of Dutch men who married between 1854 and 1922.

I conclude that a parent-offspring perspective is too narrow and misrepresents the impact of family background on the Dutch status attainment process during modernization. I base this conclusion on the finding that grandfather’s and great-grandfather’s occupational status have a substantial influence on their grandsons’ status (independent of fathers and uncles), and on the finding that the status correlation between (second) cousins is higher than would be expected had family influence been limited to that of parents. The association between the status of father and son—sometimes referred to as the intergenerational status correlation/elasticity—is often used to compare societies in terms of their openness (see, e.g., Björklund and Jäntti 2000; Ganzeboom et al. 1991; Yaish and Andersen 2012). The multigenerational model shows that this two-generational measure underestimates the influence of (extended) family background in the Netherlands during modernization. However, in terms of predicting an individual’s status (explained variance), the gain from a multigenerational model is moderate.

The second aim of this article was to gain more insight into the operation of multigenerational influence. Two important mechanisms have been proposed in the literature: influence through contact and influence without contact through durable resources and institutions. I found evidence suggesting that both mechanisms are at work. On one hand, the grandfather influence was stronger the greater the likelihood of contact between grandfather and grandson. On the other hand, a grandfather effect remained even if it was highly unlikely for a grandfather to have been in contact with his grandson. Moreover, because contact was virtually impossible for great-grandfathers, I see their effect as further support for Mare’s (2011) claim that multigenerational influence does not necessarily require contact: some privileges may endure even after the original holder has passed away.

The Netherlands modernized rapidly after 1850. Treiman (1970) and other modernization theorists have claimed that societies became more open because of these modernization processes. Therefore, durable resources were expected to have lost importance over time as a mechanism for multigenerational influence: in a more meritocratic society, status-maintaining institutions are likely to break down, and durable resources (physical capital for instance) are likely to lose ground to more perishable resources (human capital, for example). In line with this predicted change from ascription to achievement, I found that the influence of fathers, uncles, and great-grandfathers on status attainment decreased over time (although the latter was not significant).

In the same period, life expectancy increased in the Netherlands. Therefore, in the case of grandfathers, the contact mechanism was expected to have gained importance over time because contact between grandparents and grandchildren was more likely. In other words, while grandfathers were expected to lose influence because of modernization processes, they were also expected to gain influence because of the greater overlap in lives with their grandsons. The results suggest that these opposing developments cancelled each other out: grandfathers were able to retain their influence.

Because the Netherlands is a prototypical case in the sense that these developments (modernization and increasing life expectancy) occurred in many Western countries, one would expect similar findings for other Western societies. Only empirical evidence can prove whether this is true, and an exciting development in this respect is the ongoing digitization of vital registers across the world (Van Leeuwen and Maas 2010). Hopefully, it will be just a matter of time before the generations within these data are linked so that this study can be replicated.

Although the historical data used are rich in terms of allowing one to study the influence of fathers, uncles, grandfathers, and even great-grandfathers over a long period and for a large geographical area, these data have limitations. As mentioned in the Method section, a difficult issue for studies on grandfather effects is to rule out the possibility that an observed grandfather effect is partly or wholly a statistical artifact resulting from the inability to measure perfectly all the relevant resources of the intermediate generation (father, mother, uncles, and aunts) (Clark 2014). For example, the grandfather effect might (partly) reflect mother’s influence because mother’s level of resources is not directly measured and is correlated with her father-in-law’s occupational status through assortative mating (Zijdeman and Maas 2010).

Whereas most studies control only for the father’s status, an advantage of this study is that it also controls for the status of uncles. Still, these measures may not be detailed enough to filter out all effects of the intermediate generation (such as those of the mother). Chan and Boliver (2013) showed for contemporary Britain that a grandfather effect remained even after they added additional measures for parental resources (parental education, income, and homeownership). This result may offer some comfort but only to the extent that their results are generalizable to the Dutch historical context. Unfortunately, the possibilities of adding more measures of parental resources are limited when using historical data, and so the results of this study should be interpreted with some caution.

Currently, studies tend to establish whether a grandfather effect exists in a certain context. With evidence growing that such effects are indeed present in many contexts (Allingham 1967; Beck 1983; Campbell and Lee 2003, 2008, 2011; Chan and Boliver 2013, 2014; Goyder and Curtis 1977; Pohl and Soleilhavoup 1982), researchers also need to start explaining these effects. Showing that observed grandparent effects are truly the result of the mechanisms proposed in the literature helps to build confidence that grandparent effects are not just unobserved parent effects. I have taken an initial step in testing the mechanisms, although the indicators used are certainly not perfect. For example, less overlap in grandparents’ and grandsons’ lives might indicate fewer possibilities for contact but may also reflect that differences between the cohorts of the grandfather and the grandson are greater, which could inhibit the grandfather’s influence even if there was contact. Future research should advance efforts to test the mechanisms by using data with more direct measures of durable resources and of contact between grandparents and grandchildren. Contemporary studies can be designed specifically to include more direct measures. Historical studies could benefit from further digitization and linkage of historical records, which may provide information on, for example, coresidence and timing of death of family members.

Finally, the realization that a parent-offspring approach may be too limited in scope to allow an understanding of social stratification in certain contexts has prompted studies mainly of grandparents. However, this study found that the influence of uncles was almost as large as that of grandfathers and that even great-grandfathers had an impact. Without a clear idea why and under which conditions uncles or great-grandfathers can be expected to have an influence, it is difficult to claim that observed effects reflect anything more than unobserved parental or community characteristics. Therefore, we need to widen our view and develop and test theory not just on grandparents but on other extended family members as well.

## Footnotes

1 Stem families were more common, mostly among farming families, in the southeastern Netherlands than in the rest of the country. In the southeastern provinces (Drenthe, Overijssel, Gelderland, and Limburg), approximately 22 % of children were born into extended-family households, compared with 12 % in the northwestern provinces (South Holland, North Holland, Friesland, and Groningen) and 17 % in an intermediate region (Utrecht, Brabant, and Zeeland) (Kok et al. 2011).

2 These data are calculated from generation life tables (*generatie-sterftetafels*) from Statistics Netherlands (CBS). The calculations, made by Frans Van Poppel (Netherlands Interdisciplinary Demographic Institute), were received through personal communication (e-mail) on September 13, 2013.

3 Unfortunately, no earlier data are available, although approximately 70 % of the grandfathers in this study were born before 1820. Also, life expectancy is not the only factor determining the overlap in lives between generations; the age at which men have children is also relevant. Post et al. (1997), using genealogical data, concluded that overlap in lives increased mostly for children aged 0–20 from 1900 onward (more or less after my period of observation). However, they noted that their approach, too, has several shortcomings.

4 Given that farmers may form a special group, and an influential one too (see Fig. 3), I checked whether multigenerational effects are very different for those with a farming background from those without a farming background; they are not.

5 As a robustness check, I also performed the analyses without those who have no uncles. The results were not substantially different.

6 I add a fifth level when analyzing great-grandfathers.

7 Multilevel models assume that the error terms are normally distributed. The residual errors deviate somewhat from normality. However, given the findings of Maas and Hox (2004), who showed that the estimates of fixed and random effects as well as the standard errors of the fixed effects are robust against violations of the normality assumption, I do not expect serious problems.

8 To disentangle direct grandfather effects from indirect effects through the intermediate generation, I control (like most studies) for father’s status and additionally (unlike most studies) for uncles’ status. However, a concern in the literature is that such controls are not sufficient and that grandparental measures therefore reflect imperfectly measured parental effects (Chan and Boliver 2013; Clark 2014). I consider this further in the Discussion section.

9 This level of family influence is much higher than in contemporary societies (cf. Hauser and Mossel 1985; Sieben and De Graaf 2001).

10 Other possible explanations are that cousins influence one another or that uncles influence their nephews.

11 In a two-generation model (not shown), the effect of father’s status is estimated to have decreased from 0.75 in 1854 to 0.60 in 1922. This 20.0 % decrease is not much different from the 18.3 % decrease in the extended family model.

## References

[CR1] Allingham JD (1967). Class regression: An aspect of the social stratification process. American Sociological Review.

[CR2] Beck, S. H. (1983). The role of other family members in intergenerational occupational mobility. *Sociological Quarterly, 24,* 273–285.

[CR3] Bengtson VL (2001). Beyond the nuclear family: The increasing importance of multigenerational bonds. Journal of Marriage and Family.

[CR4] Björklund A, Jäntti M (2000). Intergenerational mobility of socio-economic status in comparative perspective. Nordic Journal of Political Economy.

[CR5] Björklund A, Jäntti M, Lindquist MJ (2009). Family background and income during the rise of the welfare state: Brother correlations in income for Swedish men born 1932–1968. Journal of Public Economics.

[CR6] Blau PM, Duncan OD (1967). The American occupational structure.

[CR7] Bourdieu, P., & Passeron, J.-C. (1990). *Reproduction in education, society and culture*. London, UK: Sage. (Original work published 1977)

[CR8] Bras, H. (2002). *Zeeuwse meiden. Dienen in de levensloop van vrouwen, ca. 1850–1950* [Zeeland maids: Domestic service in the life course of women, c. 1850–1950]. Amsterdam, The Netherlands: Aksant.

[CR9] Bras H, Kok J, Mandemakers K (2010). Sibship size and status attainment across contexts: Evidence from the Netherlands, 1840–1925. Demographic Research.

[CR10] Breen R, Jonsson JO (2005). Inequality of opportunity in comparative perspective: Recent research on educational attainment and social mobility. Annual Review of Sociology.

[CR11] Breen R, Luijkx R, Breen R (2004). Conclusions. Social mobility in Europe.

[CR12] Campbell C, Lee J (2003). Social mobility from a kinship perspective: Rural Liaoning, 1789–1909. International Review of Social History.

[CR13] Campbell C, Lee J (2008). Kin networks, marriage, and social mobility in late imperial China. Social Science History.

[CR14] Campbell C, Lee JZ (2011). Kinship and the long-term persistence of inequality in Liaoning, China, 1749–2005. Chinese Sociology and Anthropology.

[CR15] Chan TW, Boliver V (2013). The grandparents effect in social mobility: Evidence from British birth cohort studies. American Sociological Review.

[CR16] Chan TW, Boliver V (2014). Social mobility over three generations in Finland: A critique. European Sociological Review.

[CR17] Clark G (2014). The son also rises: Surnames and the history of social mobility.

[CR18] Collins R (1971). Functional and conflict theories of educational stratification. American Sociological Review.

[CR19] De Jonge, J. A. (1968). *De industrialisatie in Nederland tussen 1850 en 1914* [The industrialization in The Netherlands between 1850 and 1914]*.* Amsterdam, The Netherlands: Scheltema & Holkema.

[CR20] Ekamper, P., Van der Erf, R., Van der Gaag, N., Henkens, K., Van Imhoff, E., & Van Poppel, F. W. A. (2003). *Bevolkingsatlas van Nederland: Demografische ontwikkelingen van 1850 tot heden* [Population atlas of The Netherlands: Demographic developments from 1850 to the present]. Rijswijk, The Netherlands: Uitgeverij Elmar.

[CR21] Engelen, T., & Kok, J. (2003). Permanent celibacy and late marriage in the Netherlands, 1890–1960. *Population* (English ed.), 58, 67–96.

[CR22] Erikson R, Goldthorpe JH (1992). The constant flux: A study of class mobility in industrial societies.

[CR23] Erola J, Moisio P (2006). Social mobility over three generations in Finland, 1950–2000. European Sociological Review.

[CR24] Ganzeboom HBG, Luijkx R, Treiman DJ (1989). Intergenerational class mobility in comparative perspective. Research in Social Stratification and Mobility.

[CR25] Ganzeboom HBG, Treiman DJ, Ultee WC (1991). Comparative intergenerational stratification research: Three generations and beyond. Annual Review of Sociology.

[CR26] Goyder JC, Curtis JE (1977). Occupational mobility in Canada over four generations. Canadian Review of Sociology/Revue Canadienne de Sociologie.

[CR27] Hällsten M (2014). Inequality across three and four generations in egalitarian Sweden: 1st and 2nd cousin correlations in socio-economic outcomes. Research in Social Stratification and Mobility.

[CR28] Hauser RM, Mossel PA (1985). Fraternal resemblance in educational attainment and occupational status. American Journal of Sociology.

[CR29] Jencks, C., Smith, M., Acland, H., Bane, M. J., Cohen, D., Gintis, H., . . . Michelson, S. (1972). *Inequality: A reassessment of the effect of family and schooling in America*. New York, NY: Basic Books.

[CR30] Kerr C, Dunlop JT, Harbison FH, Myers CA (1960). Industrialism and industrial man: The problems of labor and management in economic growth.

[CR31] Knigge A, Maas I, Van Leeuwen MHD (2014). Sources of sibling (dis)similarity: Total family impact on status variation in the Netherlands in the nineteenth century. American Journal of Sociology.

[CR32] Knigge A, Maas I, Van Leeuwen MHD, Mandemakers K (2014). Status attainment of siblings during modernization. American Sociological Review.

[CR33] Knippenberg, H., & De Pater, B. (2002). *De eenwording van Nederland: Schaalvergroting en integratie sinds 1800* [The unification of the Netherlands: Increase in scale and integration since 1800]. Nijmegen, The Netherlands: Sun.

[CR34] Kok J, Mandemakers K (2010). A life-course approach to co-residence in the Netherlands, 1850–1940. Continuity and Change.

[CR35] Kok J, Vandezande M, Mandemakers K (2011). Household structure, resource allocation and child well-being: A comparison across family systems. Tijdschrift voor Sociale en Economische Geschiedenis.

[CR36] Lambert PS, Zijdeman RL, Van Leeuwen MHD, Maas I, Prandy K (2013). The construction of HISCAM: A stratification scale based on social interactions for historical comparative research. Historical Methods.

[CR37] Leckie G, Charlton C (2013). *runmlwin*: A program to run the *MLwiN* multilevel modeling software from within Stata. Journal of Statistical Software.

[CR38] Maas CJM, Hox JJ (2004). The influence of violations of assumptions on multilevel parameter estimates and their standard errors. Computational Statistics and Data Analysis.

[CR39] Maas, I., Van Leeuwen, M. H. D., Pélissier, J.-P., & Rébaudo, D. (2011). Economic development and parental status homogamy: A study of 19th century France. *History of the Family, 16,* 371–386.

[CR40] Mandemakers, K. (1996). *Gymnasiaal en middelbaar onderwijs: Ontwikkeling, structuur, sociale achtergrond en schoolprestaties, Nederland, ca. 1800–1986* [Pre-university and senior general secondary education: Development, structure, social background and school performance, The Netherlands, c. 1800–1968]. Amsterdam, The Netherlands: Stichting Beheer IISG.

[CR41] Mare RD (2011). A multigenerational view of inequality. Demography.

[CR42] Oosten, M. (2008). *Verleden namen: Familieverbanden uit Genlias-data* [Past names: Family ties from Genlias data] (Unpublished thesis). LIACS Leiden University, Leiden, The Netherlands.

[CR43] Pohl, R., & Soleilhavoup, J. (1982). *La transmission du statut social sur deux ou trois générations* [The transmission of social status through 2 or 3 generations]. *Economie et Statistique, 144,* 25–42.

[CR44] Post W, Van Poppel FWA, Van Imhoff E, Kruse E (1997). Reconstructing the extended kin-network in the Netherlands with genealogical data: Methods, problems, and results. Population Studies.

[CR45] Rasbash J, Charlton C, Browne WJ, Healy M, Cameron B (2013). MLwiN version 2.28.

[CR46] Schulz, W. (2013). *Careers of men and women in the 19th and 20th centuries* (Unpublished doctoral dissertation). Utrecht University, Utrecht, The Netherlands.

[CR47] Sieben IJP, De Graaf PM (2001). Testing the modernization hypothesis and the socialist ideology hypothesis: A comparative sibling analysis of educational attainment and occupational status. British Journal of Sociology.

[CR48] Smits J-P, Horlings E, Van Zanden JL (2000). Dutch GNP and its components, 1800–1913.

[CR49] Snijders TAB, Bosker RJ (1999). Multilevel analysis: An introduction to basic and advanced multilevel modeling.

[CR50] Stewart A, Prandy K, Blackburn RM (1980). Social stratification and occupations.

[CR51] Treiman DJ, Laumann E (1970). Industrialization and social stratification. Social stratification: Research and theory for the 1970s.

[CR52] Van Leeuwen MHD, Maas I (2010). Historical studies of social mobility and stratification. Annual Review of Sociology.

[CR53] Van Leeuwen MHD, Maas I, Miles A (2002). HISCO: Historical international standard classification of occupations.

[CR54] Van Poppel, F. W. A. (2012). *De familie Doorsnee tegen het licht: Anderhalve eeuw veranderingen in de Nederlandse familiestructuur* [The family Average examined: One and a half century changes in the Dutch family structure]. Utrecht, The Netherlands: Utrecht University, Netherlands Interdisciplinary Demographic Institute.

[CR55] Van Zanden JL, Van Riel A (2004). The strictures of inheritance: The Dutch economy in the nineteenth century.

[CR56] Warren JR, Hauser RM (1997). Social stratification across three generations: New evidence from the Wisconsin Longitudinal Study. American Sociological Review.

[CR57] Winer BJ, Brown DR, Michels KM (1991). Statistical principles in experimental design.

[CR58] Yaish M, Andersen R (2012). Social mobility in 20 modern societies: The role of economic and political context. Social Science Research.

[CR59] Zeng Z, Xie Y (2014). The effects of grandparents on children’s schooling: Evidence from rural China. Demography.

[CR60] Zijdeman, R. L. (2010). *Status attainment in the Netherlands, 1811–1941. Spatial and temporal variation before and during industrialization* (Unpublished doctoral dissertation). Utrecht University, Utrecht, The Netherlands.

[CR61] Zijdeman RL, Maas I (2010). Assortative mating by occupational status during early industrialization. Research in Social Stratification and Mobility.

